# Mitochondrial DNA Mutations and Epigenetic Regulation in Type 2 Diabetes Mellitus Development

**DOI:** 10.3389/bjbs.2025.15375

**Published:** 2025-11-27

**Authors:** Mei Xin Koh, Timothy Simpson, Shamsul Mohd Zain, Qasim Ayub, Hong Leong Cheah, Yan Pan, Shi Hui Cheng, Yuh-Fen Pung

**Affiliations:** 1 School of Pharmacy, Faculty of Science and Engineering, University of Nottingham Malaysia, Semenyih, Selangor Darul Ehsan, Malaysia; 2 School of Life Sciences, Faculty of Medicine and Health Sciences, University of Nottingham, Nottingham, United Kingdom; 3 Department of Pharmacology, Faculty of Medicine, University of Malaya, Kuala Lumpur, Malaysia; 4 Monash University Malaysia Genomics Platform, School of Science, Monash University Malaysia, Bandar Sunway, Selangor Darul Ehsan, Malaysia; 5 School of Biosciences, Faculty of Science and Engineering, University of Nottingham Malaysia, Semenyih, Selangor Darul Ehsan, Malaysia

**Keywords:** ethical limitations, haploid, methylation, mitochondrial dysfunction, next-generation sequencing

## Abstract

The global prevalence of type 2 diabetes mellitus (T2DM) has increased significantly over the past decade and is projected to rise further. While genetic and lifestyle factors are well-established contributors to T2DM pathogenesis, mitochondria have also gained attention as the key players. Many studies suggested that mitochondrial DNA (mtDNA) mutations and epigenetic modifications were implicated in the development and progression of T2DM. This review aimed to provide a comprehensive analysis of mtDNA mutations and epigenetic modifications associated with T2DM. Based on data from 30 published studies, a total of 117 mtDNA mutations were identified to be associated with T2DM, with D-loop region being the mutation hotspot. However, it was reported that the majority of D-loop mutations were also more frequently observed in healthy populations compared to mutations in other mtDNA regions, suggesting their potential non-pathogenic characteristic. Thus, mtDNA mutations found to be associated with T2DM but with lower occurrence in healthy populations may play a more significant role in influencing T2DM susceptibility. Regarding epigenetic modifications, mtDNA methylation was commonly reported in the D-loop and *ND6* regions across seven studies. These findings suggested that these regions may play critical roles in the regulation of mitochondrial gene expression under diabetic conditions. Lastly, this review also discussed the technical challenges and limitations of detecting mtDNA mutations and methylation changes. In addition, relevant ethical considerations surrounding mitochondrial genetic research were also addressed. In conclusion, mtDNA mutations and methylation changes could potentially serve as biomarkers for the development and progression of T2DM. These molecular modifications may offer valuable insights for early diagnosis and preventive strategies. However, further research and validation are essential to establish their clinical significance and diagnostic utility.

## Introduction

Diabetes mellitus (DM) is recognised as one of the fastest expanding global health crises. In 2024, approximately 589 million adults aged between 20 and 79 were reported to have DM, which is estimated to increase to 853 million adults by 2050 [1]. Notably, around 90% of diabetes cases globally are attributed to type 2 diabetes mellitus (T2DM) [2, 3]. Adding to this concern, T2DM incidence among individuals aged 15 to 39 rose by 56% between 1990 and 2019, further emphasising the urgent need to address this disease [4].

DM is characterised by increased blood glucose levels, often due to impaired insulin secretion from the pancreas, insulin resistance (IR) in peripheral tissues, or both [2]. Insulin is an important hormone secreted by the pancreatic *β*-cells, which facilitates glucose uptake from bloodstream into cells for energy production or storage [1]. Additionally, it plays a key role in inhibiting hepatic gluconeogenesis [5]. Therefore, reduced insulin production or sensitivity can lead to increased blood glucose levels.

It has been proposed that both genetic and epigenetic factors may influence the development of T2DM [6]. Although genome-wide association studies have identified several common genetic mutations associated with glycaemic traits (e.g., fasting glucose, fasting insulin, insulin secretion and insulin sensitivity), these account for only about 10%–20% of the variance in these traits [7]. This suggests that factors other than nuclear DNA may also play a significant role in T2DM development. In this context, mitochondrial DNA (mtDNA) is thought to affect T2DM progression, in which mutations or epigenetic modifications in mtDNA may disrupt glucose homeostasis. Although studies have been conducted to determine mtDNA mutations related to T2DM, research on mtDNA epigenetic modifications remains limited and less well-documented.

Thus, this review summarises findings from 1994 to 2024 on the link between mtDNA mutations, epigenetic modifications, and T2DM, with most studies published within the past decade. The challenges and limitations to the profiling of mtDNA mutations and methylation have also been addressed. The discovery of mutations or epigenetic variations associated with T2DM susceptibility may enable earlier diagnosis or prevention. Additionally, mtDNA epigenetic profile may serve as a valuable indicator for assessing treatment efficacy or disease progression, contributing to a significant milestone in clinical advancement.

## Mitochondrial DNA Mutations in the Development of T2DM

### Mitochondria and Mitochondrial DNA

Mitochondria are frequently referred to as the “powerhouse of the cell” due to their essential role in adenosine triphosphate (ATP) synthesis through oxidative phosphorylation. They also play several crucial metabolic roles, such as intracellular calcium homeostasis, reactive oxygen species (ROS) production, synthesis of haem and iron-sulphur clusters [8–10]. Furthermore, they play a central role in regulating programmed cell death by responding to the pro- and anti-apoptotic signals to maintain tissue homeostasis [11].

**FIGURE 1:**
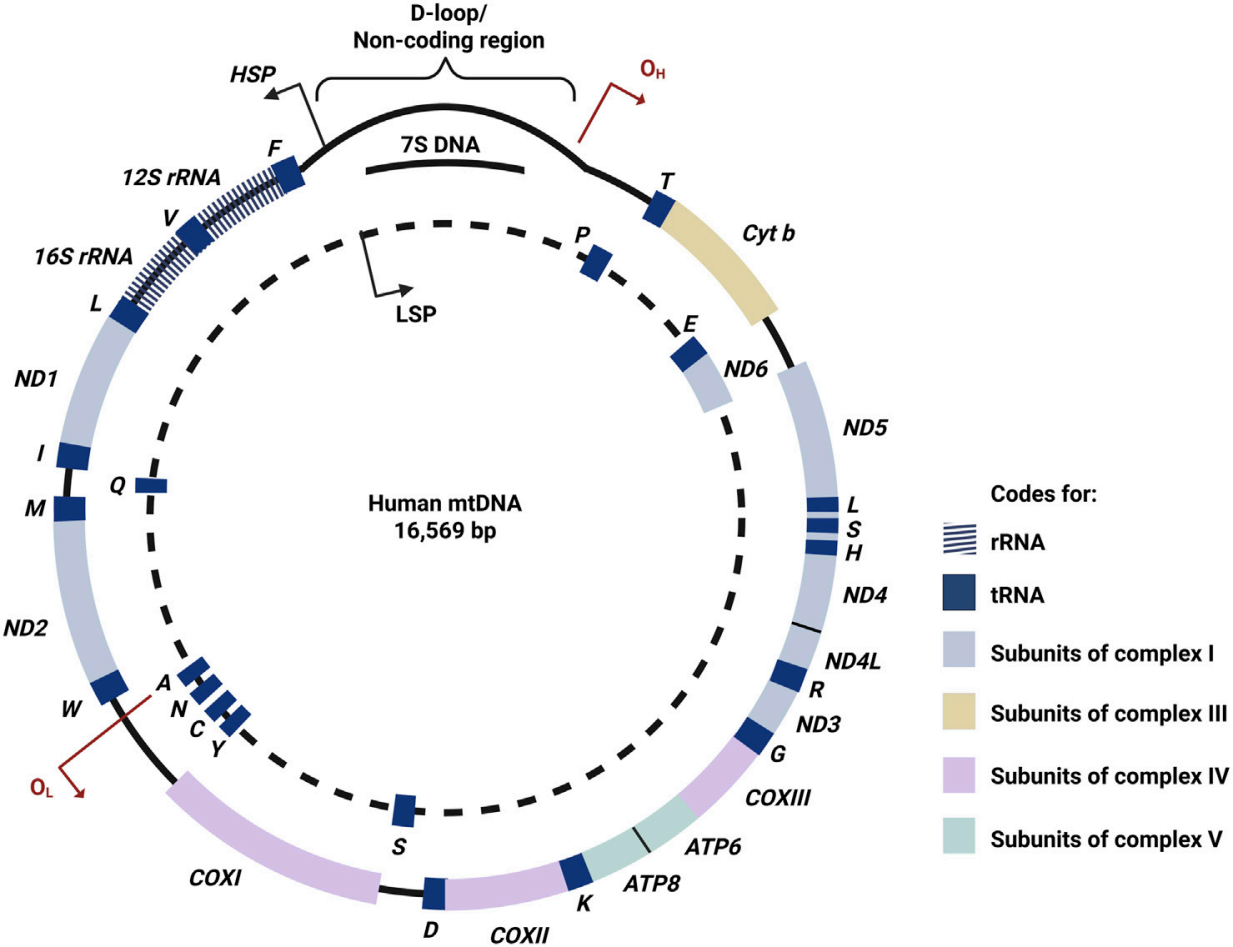
The human mitochondrial DNA. The outer circle indicates the H-strand, while the dotted line indicates the L-strand. Human mitochondrial DNA only encodes for 37 genes, namely 13 OXPHOS protein-coding genes, 22 tRNAs and 2 rRNAs [14]. H-strand: heavy strand; L-strand: light strand; O_H_: origin of H-strand synthesis; O_L_: origin of L-strand synthesis; HSP: promoter for transcription of heavy strand; LSP: promoter for transcription of light strand; bp: base pairs; D-loop: displacement loop: rRNA: ribosomal RNA; *ND*: NADH dehydrogenase; *COX*: cytochrome c oxidase; *ATP*: ATP synthase; *Cyt b*: Cytochrome b; tRNAs for *F*: Phenylalanine; *V*: Valine; *L*: Leucine; *I*: Isoleucine; *Q*: Glutamine; *M*: Methionine; *W*: Tryptophan; *A*: Alanine; *N*: Asparagine; *C*: Cysteine; *Y*: Tyrosine; *S*: Serine; *D*: Aspartic acid; *K*: Lysine; *G*: Glycine; *R*: Arginine; *H*: Histidine; *E*: Glutamic acid; *T*: Threonine; *P*: Proline; OXPHOS: oxidative phosphorylation.

Mitochondria are distinct from other cytoplasmic organelles as they contain their own DNA, which encodes essential RNAs and proteins [12]. Human mtDNA encodes only 37 genes due to the evolutionary loss or transfer of most mitochondrial genes to nuclear DNA [13]. Among these, 22 genes encode for tRNAs, 2 rRNAs (12S and 16S) and 13 are protein-coding genes (Figure 1) [13]. Unlike traditional Mendelian genetics, mtDNA is inherited maternally as a haploid molecule, allowing mutant mtDNA to accumulate and be passed from mother to offspring [15, 16].

Human mtDNA is a circular, polycistronic, double-stranded molecule made up of 16,569 base pairs (bp) and lacks introns (Figure 1) [17]. The nucleotide content of each strand differs, whereby the light strand (L-strand) is rich in cytosine, while the heavy strand (H-strand) is rich in guanine [17]. Next, mtDNA possesses a triple-stranded displacement loop structure known as displacement loop (D-loop), which is a non-coding region that acts as the promoter for both strands [18]. The D-loop regulates mtDNA replication, optimising the mtDNA copy numbers according to the cellular energy demands [19, 20].

### Oxidative Stress and Mitochondrial Dysfunction

In mitochondria, ROS (e.g., superoxide radicals, hydrogen peroxide and hydroxyl radicals) are inevitably produced due to the continuous oxidative phosphorylation activity, accounting for approximately 90% of cellular ROS [21, 22]. Under normal circumstances, the cells possess defence mechanisms to counteract ROS generation [23]. For instance, the cells possess antioxidant enzymes that neutralise ROS, including superoxide dismutase, catalase, glutathione reductase, glutathione peroxidase, thioredoxin reductase, thioredoxin and peroxiredoxin [23, 24]. In addition to enzymatic antioxidants, mitochondria contain low molecular weight antioxidants (e.g., coenzyme Q) and repair mechanisms that help mitigate oxidative damage [23]. However, excessive ROS production can still be induced by stress factors, leading to excessive electrons being transferred to oxygen in the electron transport chain without ATP production [25].

Excessive ROS production has been implicated in causing oxidative damage to DNA, contributing to the development of various diseases [26]. It has been strongly associated with mitochondrial dysfunction, resulting in increased mutation rates, reduced mitochondrial biogenesis and accelerated aging [25, 27]. The ROS-induced mitochondrial dysfunction further suppresses ATP production while increasing ROS production, creating the ‘vicious cycle’ that ultimately leads to insulin resistance and onset of DM [25]. Additionally, excessive ROS disrupts the phosphorylation of insulin receptor substrate proteins by serine kinases, which is a critical initial step in the insulin signalling pathway [28].

In turn, insulin resistance or DM can further disrupt the mitochondrial metabolism, causing reduced insulin secretion from pancreatic *β*-cells, enhanced mitochondrial permeability transition, or increased mitochondrial apoptosis [29, 30]. Studies conducted on the relationship between mitochondrial mutations, mitochondrial dysfunctions and T2DM supported the involvement of mitochondria in T2DM development [31].

### Mitochondrial DNA Mutations and T2DM

Mutation is a permanent and heritable alteration in DNA that often leads to changes in protein function [32]. It can also affect the structure and function of non-coding RNAs such as tRNA and rRNA [33]. Mutations may happen spontaneously or be induced by ROS. MtDNA is particularly susceptible to mutation due to its haploid nature and the close proximity to ROS production site, as aforementioned [21, 34]. Additionally, mtDNA lacks protective histones, leaving it more exposed to damage induced by ROS or other mutagens [21]. Moreover, unlike nuclear DNA, mtDNA lacks sufficient repair mechanisms to correct mutation and maintain normal mitochondrial function [35]. This susceptibility of mtDNA to mutation has led to the concept of a ‘vicious cycle’, in which initial ROS-induced mitochondrial dysfunction triggers further ROS production, subsequently exacerbating mitochondrial damage and dysfunction [21].

Recent studies have identified mtDNA mutations as potential contributors to the development of DM. Several mtDNA mutations associated with DM in various populations have been reported (Table 1). Beyond identifying mutations detected in diabetic individuals, it is equally important to determine whether these mutations also occur frequently in healthy individuals. Therefore, gnomAD 3.1 and Helix frequencies were included, which acted as the indicators of whether specific mutations are commonly found in healthy individuals, rather than exclusively in diabetic individuals [37–39]. In most cases, a mutation that appears at high frequency in healthy populations is likely a non-pathogenic polymorphism or mutation hotspot. Thus, the potentially significant mutations were highlighted in bold in Table 1, as they might be the more reliable indicators for different forms of DM.

**TABLE 1:** Summary of mitochondrial DNA mutations associated with diabetes or insulin resistance.

mtDNA region	Mutation^a^	No. of haplogroups reported from PhyloTree 17.0 [36]	gnomAD 3.1 frequency (%)^b^ [37, 38]	Helix Frequency (%)^c^ [37, 39]	Patient report from Mitomap [37]	Population(s) identified by studies	Type(s) of DM/IR identified	References
D-loop	A16051G	25	2.529	2.580	N/A	Bangladeshi	T2DM	[40]
	T16093C	57	5.311	2.590	N/A	Uyghur; Chinese; not specified	T2DM/MIDD	[41–44]
	T16126C	21	14.321	17.653	N/A	Bangladeshi; not specified	T2DM	[40, 44]
	G16129A	93	11.526	7.282	N/A	Bangladeshi	T2DM	[40]
	C16186T	3	1.386	NR	N/A	Arab	T2DM	[45]
	**T16189C**	117	**24.856**	**NR**	Reported DM	Italian; bangladeshi; caucasian; Chinese; not specified	T2DM	[40, 41, 46–48]
	C16223T	33	39.409	18.185	N/A	Bangladeshi; not specified	T2DM	[40, 49]
	C16270T	18	8.836	7.629	N/A	Moroccan	T2DM	[50]
	G16274A	36	1.381	1.079	N/A	Arab	T1DM/T2DM	[45]
	C16292T	24	3.466	2.477	N/A	Arab	T2DM	[45]
	C16294T	31	14.190	10.619	N/A	Arab	T2DM	[45]
	T16311C	147	21.850	17.076	N/A	Bangladeshi	T1DM	[40]
	G16319A	39	6.228	4.645	N/A	Bangladeshi	T2DM	[40]
	C16320T	25	4.753	1.710	N/A	Moroccan	T2DM	[50]
	T16519C	High	65.725	64.287	N/A	Italian; not specified	T2DM	[41, 46]
	T58C	1	0.025	0.067	N/A	Not specified	T2DM	[41]
	C150T	76	16.628	10.078	N/A	Arab	T2DM	[45]
	C151T	42	3.544	1.553	N/A	Not specified	T2DM	[41]
	T195C	124	27.606	17.602	N/A	Arab	T2DM	[45]
	568 poly C	-	-	-	N/A	Not specified	MIDD	[41, 51]
12S RNA	T1189C	3	4.005	5.965	N/A	Not specified	T2DM	[49]
	C1310T	2	0.034	0.048	N/A	Japanese; not specified	T2DM	[41, 52]
	**A1382C**	-	**0.066**	**0.079**	Reported T2DM susceptibility	Not specified	T2DM	[41]
	T1420C	2	0.353	0.139	N/A	Not specified	T2DM	[49]
	A1438G	14	95.610	96.891	N/A	Japanese	T2DM	[52]
16S RNA	G1719A	28	4.033	5.127	N/A	Arab	T2DM	[45]
	A1811G	7	8.794	12.126	N/A	Not specified	T2DM	[49]
	G1888A	14	6.360	9.489	N/A	Arab	T2DM	[45]
	T2667C	-	0.009	0.009	N/A	Not specified	T2DM	[49]
	A2706G	10	73.949	63.340	N/A	Uyghur	T2DM	[43]
	T3027C	8	0.236	0.366	N/A	Not specified	T2DM	[49]
	A3156G	-	0.007	0.007	N/A	Not specified	T1DM/T2DM	[41]
	T3200C	2	0.090	0.055	N/A	Not specified	T2DM	[41]
tRNA leu	**A3243G**	-	**0.000**	**0.001**	Confirmed pathogenic MELAS/MIDD	Chinese; Uyghur; Japanese; Indian; not specified	T1DM/T2DM/MIDD/GDM	[41, 43, 53–56]
	**C3254A**	1	**0.243**	**0.041**	Reported GDM	Singaporean; not specified	GDM	[41, 57]
	**C3256T**	-	**NR**	**NR**	Confirmed likely pathogenic MELAS	Not specified	T2DM/MIDD	[41, 44]
	**T3264C**	-	**0.000**	**0.001**	Reported DM	Japanese; not specified	MIDD/MDM	[41, 58]
	**T3271C**	-	**0.000**	**0.000**	Confirmed pathogenic MELAS/DM	Chinese	T2DM/MIDD	[56]
	T3290C	6	0.145	0.096	N/A	Not specified	T2DM/MIDD	[41, 44]
	A3302G	-	0.000	0.000	N/A	Not specified	PCOS-IR	[41]
ND1	**G3316A**	12	**0.457**	**0.495**	hg D1, D2, M33, R30 marker^d^; reported DM	Indonesian; Mizo; not specified	T2DM	[41, 44, 59, 60]
	G3357A	2	0.044	0.059	N/A	Not specified	T1DM/T2DM	[41]
	C3375A	-	NR	NR	N/A	Not specified	T1DM/T2DM	[41]
	**T3394C**	8	**0.911**	**1.085**	hg M9 marker^d^; reported DM	Chinese; Indonesian; Mizo; not specified	T1DM/T2DM	[41, 44, 48, 59, 60]
	**T3398C**	7	**0.239**	**0.270**	Reported GDM	Singaporean; not specified	GDM	[41, 57]
	**A3399T**	2	**0.005**	**0.009**	Reported GDM	Singaporean; not specified	GDM	[41, 57]
	G3483A	6	0.190	0.173	N/A	Not specified	T2DM	[44]
	T3548C	4	0.034	0.036	N/A	Not specified	MIDD	[41]
	C3970T	2	0.588	0.581	N/A	Mizo	T2DM	[60]
tRNA Ile	G4284A	-	0.007	0.001	N/A	Not specified	MIDD	[41]
tRNA Met	A4435G	2	0.041	0.066	N/A	Chinese	T2DM	[56]
	C4467A	-	NR	NR	N/A	Chinese	T2DM	[56]
ND2	G4491A	8	0.298	0.384	N/A	Not specified	T2DM	[41]
	A4769G	6	98.387	97.684	N/A	Arab	T2DM	[45]
	A5178C	-	NR	NR	N/A	Japanese; not specified	T1DM/T2DM	[41, 49]
tRNA Trp	A5514G	1	0.005	0.015	N/A	Chinese	T2DM	[56, 61]
tRNA Ala	**T5587C**	1	**0.012**	**0.015**	Reported MIDD	Chinese	T2DM	[56]
	T5628C	1	0.096	0.086	N/A	Chinese	MDM	[62]
	A5655G	2	NR	NR	N/A	Chinese	T2DM	[56]
tRNA Cys/tRNA Tyr	A5826G	1	NR	NR	N/A	Chinese	T2DM	[63]
COXI	G5913A	2	0.530	0.633	N/A	Not specified	MIDD	[41]
	C7028T	2	74.940	63.008	N/A	Uyghur	T2DM	[43]
tRNA Ser	C7502T	-	0.009	0.003	N/A	Chinese	T2DM	[56]
	T7505C	-	NR	0.001	N/A	Chinese	T2DM	[56]
tRNA Lys	8,281 9bp	19	NR	NR	N/A	Not specified	MIDD	[41, 51]
	A8296G	3	0.044	0.048	N/A	Chinese	MIDD	[56]
	G8313A	-	0.000	NR	N/A	Indian; not specified	T2DM	[41, 53]
	A8344G	-	0.000	0.000	N/A	Not specified	T2DM/GDM	[41]
	A8348G	2	0.094	0.151	N/A	Not specified	MIDD	[41]
	T8356C	-	0.000	0.000	N/A	Not specified	T2DM	[41]
ATP8	C8478T	2	0.486	0.704	N/A	Not specified	MIDD	[41]
	T8414G	1	NR	NR	N/A	Uyghur; not specified	T2DM	[41, 43]
	C8393T	2	0.028	0.040	N/A	Not specified	MIDD	[41]
Region between ATP8 and ATP6	T8551C	-	0.018	0.020	N/A	Not specified	MIDD	[41]
	**C8561G**	-	**NR**	**NR**	Reported DM	Not specified	T2DM	[41]
ATP6	A8701G	10	30.319	8.753	N/A	Mizo	T2DM	[60]
	A8860G	4	99.381	98.774	N/A	Not specified	MIDD	[41]
COXIII	**G9267C**	-	**NR**	**NR**	Reported MIDD	Not specified	MIDD	[41]
	T9540C	1	30.433	8.791	N/A	Mizo	T1DM	[64]
	A9827G	-	NR	NR	N/A	Not specified	T1DM	[41]
tRNA Gly	**T10003C**	1	**0.025**	**0.068**	Reported MIDD	Chinese; not specified	T2DM/MIDD	[41, 56]
	A10055G	-	0.004	0.004	N/A	Chinese	T2DM	[56]
ND3	A10398G	24	41.848	25.166	hg L, M marker^d^	Mizo; not specified	T2DM	[44, 64]
	C10400T	1	5.275	4.611	N/A	Mizo	T2DM	[64]
tRNA Arg	T10463C	5	5.802	8.966	N/A	Arab	T2DM	[45]
ND4L	T10873C	1	30.495	8.833	N/A	Mizo	T2DM	[64]
	G11696A	6	0.099	0.135	N/A	Chinese	T2DM	[65]
	G11914A	48	10.858	5.329	N/A	Arab	T2DM	[45]
	**A12026G**	4	**0.108**	**0.096**	Reported DM	Japanese; not specified	T2DM	[41, 52]
tRNA Ser	C12237T	2	0.021	0.022	N/A	Chinese	T2DM	[61]
	**C12258A**	-	**NR**	**NR**	Confirmed likely pathogenic MIDD	Not specified	MIDD	[41, 66]
tRNA Leu	A12230G	-	0.000	0.000	N/A	Chinese	T2DM	[56]
	A12308G	3	15.539	19.993	Reported not pathogenic in hg K and U^d^	Chinese	T2DM	[56]
ND5	C12633A	4	1.271	1.893	N/A	Arab	T2DM	[45]
	C12705T	2	48.879	18.024	N/A	Mizo	T2DM	[64]
	G13368A	12	5.779	8.901	N/A	Arab	T2DM	[45]
	G13590A	11	10.851	3.257	N/A	Arab	T2DM	[45]
ND6	G14364A	12	0.489	0.614	N/A	Arab	T2DM	[45]
	T14502C	7	0.156	0.195	N/A	Chinese	T2DM	[54]
	**T14577C**	4	**0.089**	**0.179**	Reported MIDD	Not specified	T2DM	[41, 49]
tRNA Glu	**A14692G**	1	**0.002**	**0.009**	Reported MIDD	Chinese	MIDD	[67]
	**A14693G**	7	**0.193**	**0.187**	Reported MELAS	Chinese; not specified	T2DM/MIDD	[41, 48]
	**T14709C**	-	**0.000**	**NR**	Confirmed likely pathogenic MIDD	Not specified	T2DM	[41, 44]
Cytb	C14766T	4	70.679	58.525	N/A	Not specified	T1DM/T2DM	[49]
	T14783C	2	5.535	4.885	N/A	Mizo	T2DM	[64]
	G15043A	7	7.876	7.868	N/A	Mizo	T2DM	[64]
	G15148A	10	0.333	0.429	N/A	Arab	T2DM	[45]
	G15301A	7	24.462	8.104	N/A	Mizo; Arab	T2DM	[45, 64]
	A15326G	9	99.342	98.973	N/A	Not specified	MIDD	[41]
	A15607G	4	5.558	8.735	N/A	Arab	T2DM	[45]
	A15746G	2	0.236	0.246	N/A	Not specified	T2DM	[41]
tRNA Thr	**G15897A**	-	**0.000**	**NR**	Reported MID	Chinese	T2DM/MIDD	[56, 68]
	A15901G	1	0.005	0.002	N/A	Japanese	T2DM	[69]
	A15924G	31	4.115	5.064	N/A	Chinese; not specified	T2DM	[44, 56]
	C15926T	-	0.012	0.028	N/A	Japanese	T2DM	[69]
	G15927A	7	0.709	0.920	N/A	Chinese; not specified	T2DM	[44, 56]
	G15928A	6	5.609	8.707	N/A	Arab; not specified	T2DM	[44, 45]

^a^ “TxxxxC” represents mitochondrial DNA, point mutation which occurs at position xxxx, where “T” is being replaced by “C”. “xxxx poly C″ represents that the polycytidine tract is variable in length at position xxxx. “xxxx xbp” represents mitochondrial DNA, insertion mutation which occurs at position xxxx, where a repeated sequence of x base pairs is inserted. All mutations positions are reported according to the revised Cambridge Reference Sequence (rCRS, NC_012920.1). Haplogroup associations and presence in healthy individuals were determined using PhyloTree 17.0 and Mitomap. Mutations potentially significant in diabetes are highlighted in bold.

^b^ The gnomAD, 3.1 frequency refers to the variant frequency in healthy population based on the mitochondrial dataset from the Genome Aggregation Database (gnomAD v3.1). The frequency is derived from 70% Eurasian lineage (N), 25% African lineage (L) and 5% Asian lineage (M).

^c^ The Helix frequency refers to the variant frequency in healthy population based on the Helix population database. This frequency is derived from 91.2% Eurasian lineage (N), 4.2% African lineage (L) and 4.6% Asian lineage (M).

^d^ Mitochondrial haplogroup (hg) denotes specific maternal mtDNA, lineages. These markers indicate mtDNA, variants commonly found in specific maternal lineages and may represent population-specific polymorphisms rather than pathogenic mutations.

Haplogroup distribution, variant frequencies in healthy populations (as reported in public databases), clinical reports and examples of studies that observed these mutations in different populations were listed. T1DM: Type 1 diabetes mellitus; T2DM: Type 2 diabetes mellitus; MIDD: maternally inherited diabetes & deafness/mitochondrial diabetes; MID: mitochondrial inherited diabetes; MELAS: mitochondrial encephalopathy, lactic acidosis and stroke-like episodes; GDM: gestational diabetes mellitus; PCOS-IR: insulin resistance in polycystic ovary syndrome; NR: not reported; hg: mitochondrial haplogroup (e.g., D1, D2, M33, R30, M9, L, M, K, U).

It was observed that while a majority of the mutations listed were located in the D-loop region, their frequencies in healthy populations based on both databases, were relatively higher compared to mutations in other regions of the mitochondrial genome. This suggests that many of the D-loop mutations linked to T2DM may represent random, non-pathogenic variants, rather than disease-causing mutations. This interpretation is supported by the fact that D-loop, which plays a key role in regulating mtDNA replication and transcription, is the most variable region of the mitochondrial genome in both healthy and diseased individuals [41]. Nevertheless, it is possible that D-loop mutations may still disrupt mtDNA replication, leading to mtDNA depletion and subsequent mitochondrial dysfunction, contributing indirectly to disease pathology [70].

While D-loop mutations are frequently observed in healthy individuals and likely non-pathogenic, certain other mutations may demonstrate a stronger disease association. For instance, one commonly affected site that is rarely found in healthy population is the tRNA^Leu^ gene, where adenine is substituted with guanine at nucleotide position 3243 (m.A3243G) [71, 72]. This mutation is also referred to as the mitochondrial encephalopathy, lactic acidosis and stroke-like episodes (MELAS) mutation, as it was initially identified in individuals with these clinical manifestations in 1990 [73]. This mutation disrupts the proper folding of tRNA molecule by interfering with key hydrogen bonds between A14 and U8, resulting in increased structural openness, reduced stability and reduced aminoacylation [74, 75]. To compensate, the mutant tRNA may abnormally form dimers with other mutant tRNA molecules, which further reduces the aminoacylation rate [74]. As a result, amino acids may be incorrectly incorporated or entirely omitted during mitochondrial translation, producing defective mitochondrial proteins and ultimately impairing mitochondrial function.

In summary, beyond identifying mutations present in diabetic individuals, it is equally important to assess whether these mutations also occur frequently in healthy populations. This distinction may further refine the understanding of specific genetic factors underlying mitochondrial dysfunction in diabetes, thereby contributing to more efficient risk detection. In this context, oxidative stress represents one of the key factors driving mtDNA mutations due to elevated levels of ROS in mitochondria. Thus, addressing oxidative stress and its effect on mtDNA mutations may offer promising strategies for the prevention or management of mitochondrial dysfunction in T2DM.

## Mitoepigenetics in the Development of T2DM

**FIGURE 2:**
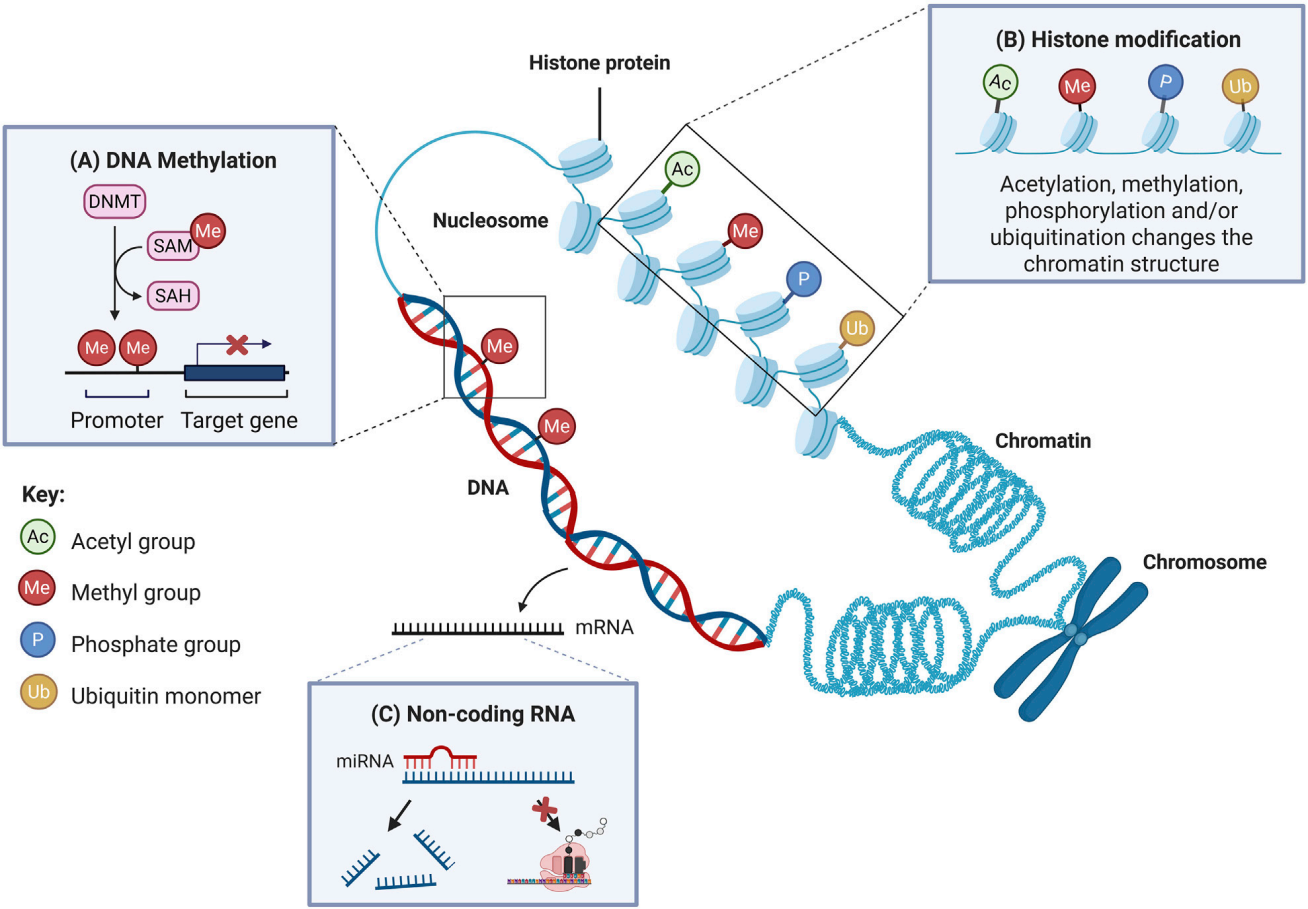
The primary mechanisms of epigenetics involve DNA methylation, histone modifications and regulation by non-coding RNAs. **(A)** DNA methylation involves the addition of a methyl group to the fifth carbon of a cytosine residue. When methylation takes place at gene promoter regions, it usually leads to downregulation of gene expression. **(B)** Histone modification refers to post-translational modification of histone proteins, such as covalent addition of acetyl, methyl, phosphate groups or ubiquitin monomers. These modifications alter the chromatin structure and affect transcriptional activity. Histone acetylation and phosphorylation typically promote transcription, while methylation is often linked to transcriptional repression. Ubiquitination can either activate or suppress transcription, depending on the context. **(C)** Non-coding RNAs, such as miRNAs, regulate gene expression post-transcriptionally. When binding to target mRNAs, miRNAs either inhibit translation or promote mRNA degradation, thus silencing gene expression. DNMTs: DNA methyltransferases; SAM: S-adenosylmethionine; SAH: S-adenosylhomocysteine. Adapted and modified with permission from Low et al. [77]. Additional details from Liu et al. [78].

Epigenetics is the study of variations in gene expression that occur without DNA sequence alterations [76]. Key epigenetic mechanisms include DNA methylation, histone modifications and regulation of gene expression by non-coding RNAs (Figure 2) [79]. These mechanisms play important roles in regulating gene expression at the transcriptional, post-transcriptional and translational levels [80]. It is proposed that epigenetics not only occurs in nuclear DNA, but it also affects mtDNA, in which the phenomenon is known as “mitoepigenetics” [81]. The processes underlying mitoepigenetics have not been as thoroughly explored as those governing nuclear DNA, mainly due to the absence of histones in mitochondria [81, 82].

**FIGURE 3:**
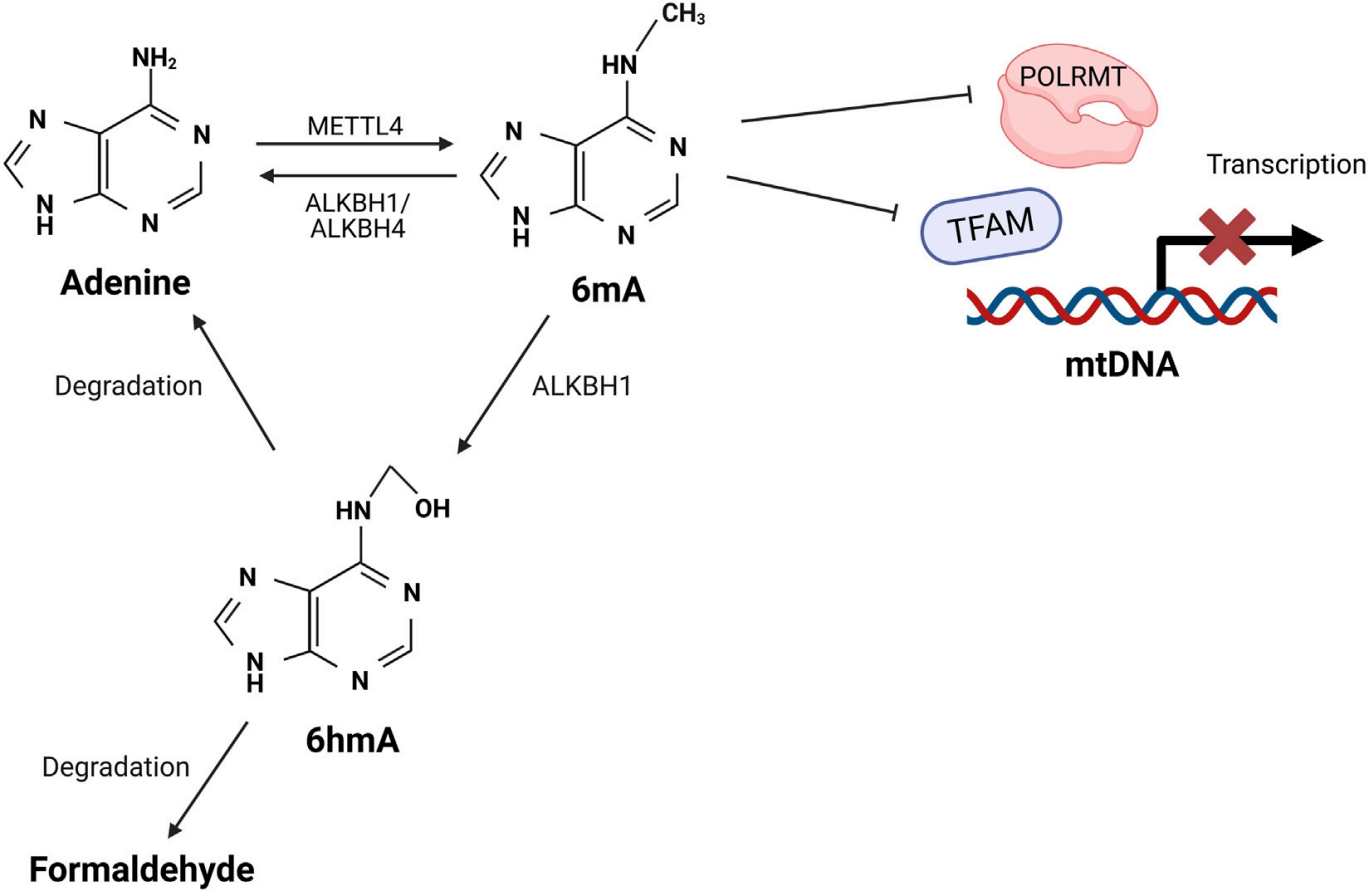
Proposed mechanisms of mtDNA methylation. Methyltransferase METTL4 catalyses the formation of 6mA on mtDNA, which interferes with the binding of TFAM and POLRMT with mtDNA, thus preventing the assembly of transcription initiation complex. This inhibition reduces mtDNA transcription and impairs mitochondrial function. The 6mA can be removed through oxidative demethylation mediated by ALKBH1 or AKLBH4, which restores adenine to its unmethylated form. Alternatively, ALKBH1 can oxidise 6mA to an unstable intermediate 6hmA, which rapidly degrades into formaldehyde and adenine [87–91]. METTL4: methyltransferase 4 MTA70; A: adenine; 6mA: N6-methyladenine; TFAM: transcription factor A; POLRMT: mitochondrial RNA polymerase; ALKBH: alpha-ketoglutarate-dependent dioxygenase homolog; 6hmA: 6-hydroxymethyladenine.

While some studies suggest that methylation is a distinct feature of nDNA and absent in mtDNA, others provide evidence supporting its presence in mtDNA [83–86]. Emerging studies have identified N^6^-methyldeoxyadenosine (6mA) as an alternative form of mtDNA methylation, mediated by methyltransferase 4 MTA70 (METTL4), alongside the more commonly studied 5-methylcytosine (5-mC) (Figure 3). Notably, the deletion of METTL4 has been shown to reduce 6mA levels in mtDNA [87]. However, studies have reported conflicting findings regarding the primary methylation sites in mtDNA, with evidence suggesting involvement of both CpG and non-CpG sites [92–95]. While the majority of studies suggest that methylation primarily occurs at CpG sites, Patil et.al. [93] reported that non-CpG sites are the main targets of methylation in mtDNA. Despite these conflicting findings, one consistent observation is that methylation occurs more frequently on the L-strand compared to the H-strand [93–96].

Several studies have suggested that mtDNA methylation serves as a protective mechanism against oxidative damage, which could potentially be induced by high blood glucose concentrations in patients with T2DM [97, 98]. The frequency and sites where mtDNA methylation have been detected in the progression of T2DM and related disorders are summarised in Table 2. An *in vitro* study showed that diabetic condition increased mtDNA methylation by 1.5- to 3-fold at various regions, including the D-loop, *Cytb*, *ND6* and *COXII* [99]. Similar findings have also been reported by several *in vivo* studies. For instance, Kowluru (2020) found higher levels of D-loop methylation in the retinal microvasculature of T2DM rats [100]. Meanwhile, another study reported significant methylation at the *ND1*, *ND2*, *ND6*, *CYTB* and *COX1* regions in diabetic mice [95]. The latter study also found significant higher methylation of *ND6* in T2DM human subjects compared to healthy controls [95]. Notably, IR subjects displayed significant 4.6-fold increase in DNA methylation compared to insulin-sensitive subjects [103].

**TABLE 2:** mtDNA methylation studies in T2DM or related disorders.

Experimental model	mtDNA Region(s) investigated	Key Observation(s)	References
**Cell culture**			
High glucose (20 mM) treated *in vitro* cultured bovine retinal endothelial cells and human retinal microvasculature	D-loop*, Cytb, ND6* and *COXII*	Increased methylation, decreased mtDNA transcription and increased DNMT1 binding were observed at D-loop. Methylation levels were significantly higher at D-loop compared to *Cytb* and *COXII* regions (*p* < 0.05). This resulted in significant inhibition of the mitochondrial gene expression critical for electron transport chain activity (*p* < 0.05)	[99]
**Animal model**			
Hepatic mtDNA from *db/db* mice	13 mtDNA-encoded genes	Significant methylation was observed at *ND2*, *ND5*, *ND6*, *COX1* and *ATP8* regions, with *ND6* region showing the highest methylation level under diabetic conditions due to enhanced mitochondrial translocation of DNMT1 (*p* < 0.05)	[95]
Retinal microvasculature from T2DM, T1DM diabetes rat models, and high-fat diet rat models	D-loop	D-loop methylation level was higher in the T2DM group compared to the T1DM or high-fat diet group	[100]
**Clinical studies**			
Peripheral leukocytes from 39 obese and 39 non-obese human subjects	*ND6*	Significant increased *ND6* methylation was observed in T2DM subjects compared to healthy controls. *ND6* methylation level was inversely correlated with *ND6* expression and positively correlated with metabolic parameters including body mass index, fasting glucose, fasting insulin and insulin resistance index (*p* < 0.05)	[95]
Buccal swabs from 69 young caucasian individuals	D-loop	D-loop methylation was significantly higher in overweight females than lean females. Increased methylation was associated with reduced mtDNA copy number, and mtDNA copy number showed a negative correlation with BMI in females (*p* < 0.05)	[101]
Leukocytes from fasting blood samples of 8 lean and 32 obese/overweight participants	D-loop and *ND6*	D-loop and *ND6* methylation levels were significantly correlated with insulin resistance indices (*p* < 0.05)	[102]
Leukocytes from fasting blood samples of 40 participants without diabetes or cardiovascular disease	D-loop	A 5.2-fold increase of D-loop methylation was observed in obese than in lean subjects; A 4.6-fold increase of D-loop methylation was observed in insulin-resistant than in insulin-sensitive subjects	
Liver biopsies from 45 NAFLD patients and 18 with near-normal histology	D-loop*, ND6* and *COX1*	Methylated/unmethylated *ND6* ratio was significantly correlated with NAFLD activity score, whereas D-loop and *COX1* methylation were not correlated with disease severity	

Limited clinical research has been conducted to characterise the mtDNA methylation profile in T2DM subjects. However, since T2DM is often associated with obesity or being overweight, studying the methylation profile in such individuals may provide valuable insights. For example, a clinical study reported that increased methylation at specific CpG sites in the D-loop was significantly (*p* = 0.003) associated with body mass index (BMI) percentiles >85th in female subjects [101]. Similar observations were made in an earlier mixed-gender study, in which a 5.2-fold increase in D-loop methylation was observed in obese subjects compared to lean subjects [103]. This was further supported by a subsequent study that revealed a drastic elevation of methylation levels at *ND6* and D-loop regions as BMI increased [102].

Research has also demonstrated the common coexistence of non-alcoholic fatty liver disease (NAFLD) and T2DM. Specifically, NAFLD increases the risk of T2DM by 2- to 5-fold, with about 59.67% of T2DM patients also having NAFLD [104, 105]. This connection suggests that the methylation profile in individuals with NAFLD may be linked to T2DM. For instance, one study reported a significant positive association (*p* < 0.04) between the methylated-to-unmethylated ratio of *mt*-*ND6* and the severity of NAFL, with methylation levels increasing from 20.6% in simple steatosis to 28.4% in non-alcoholic steatohepatitis [106].

In summary, increased mtDNA methylation is a response to various stress factors in disease states to protect mtDNA from damage and potential mutations. Several findings have indicated that mtDNA methylation profile contains relevant information about body composition and the associated risk of developing diseases such as T2DM. Given its roles in gene expression regulation, the potential of epigenetics in disease development or prevention warrants further research. Consequently, these epigenetic profiles might be a useful indicator for early molecular detection or prevention, as they are often detectable in the early stages of disease progression [107].

## Limitations and Challenges in Sequencing Mitochondrial DNA Mutations and Mitochondrial Epigenetic Modifications

While research on mtDNA mutations and epigenetic modifications has advanced the understanding of mitochondrial regulation in T2DM, the accurate detection of these changes remains technically challenging. To date, hundreds of thousands of human mitogenomes have been sequenced using traditional Sanger methods, as well as a range of modern high-throughput sequencing platforms. Meanwhile, studies on the mitoepigenome remain limited but are getting increasing attentions with advancements in sequencing technologies. These sequencing technologies typically involve several steps including DNA isolation, library preparation and sequencing that uses various chemistries, flow-cells and detection systems utilizing base-specific color-coded fluorescence, light emissions, current, or ions changes. This is followed by bioinformatics analysis and each stage of the process presents unique challenges, making mtDNA sequencing particularly challenging.

### Mitochondrial DNA Isolation and Purification

The initial step of isolating and enriching pure mtDNA relative to nuclear DNA remains challenging. Contamination with nuclear DNA, including mitochondrial pseudogenes in the lysate, can introduce artifacts during mtDNA analysis, particularly with short-read lengths [108]. Various conventional isolation techniques have been used, such as differential centrifugation, density gradient centrifugation, magnetic bead-based and mitochondrial isolation followed by DNA extraction [109–111]. Several commercial kits for direct mtDNA extraction are widely available. One study has also demonstrated a novel approach for mtDNA isolation and enrichment using a plasmid isolation kit, followed by additional purification with solid-phase reversible immobilisation on paramagnetic beads and limited polymerase chain reaction (PCR) amplification [112]. However, a very high cell number, typically ranging from 5 to 17 million cells is required from cell culture and patient samples to obtain sufficient mtDNA yield for sequencing, regardless of the method used [110, 112]. This requirement may be impractical for certain studies, particularly those involving clinical samples, which are often scarce.

Other options include DNA extraction followed by target enrichment of mtDNA by either long-range PCR amplification or complementary oligonucleotide probe hybridization. Targeted amplification may be suitable for direct sequencing but sequencing artefacts may be introduced during the DNA amplification, which may lead to false positive or negative results [113]. Moreover, mtDNA enrichment via PCR amplification is unsuitable for methylation sequencing, as PCR does not preserve methylation marks (such as 5-mC), compromising accurate quantification of mtDNA methylation [112, 114]. A mtDNA isolation method capable of providing high yield and enrichment with minimal or no amplification is ideal for downstream methylation sequencing and improvements are still needed in DNA extraction methods and protocols for investigation of epigenetic markers in an unbiased manner.

### Sequencing Methods

Sequencing technologies are categorised into first-, second- and third-generation methods, with the latter two often referred as next-generation sequencing (NGS) [115]. The selection of an appropriate sequencing technology depends on the specific research question being asked. Different sequencing methods and platforms exhibit varying degrees of error rates, ranging from 0.1% to 5% as compared to the lower error rate of Sanger sequencing (0.001%) [113, 116–122]. Although these error rates might seem to be negligible, their cumulative effect can become significant given the vast size of the human nuclear genome [113].

Sanger sequencing (first-generation) is considered the gold standard due to its high accuracy despite its short-read limitations, but it is expensive compared to the newer technologies that have gained popularity, particularly for human whole genome sequencing [113]. These technologies have been increasingly integrated into clinical diagnostic practices, enabling a holistic analysis of genetic variants in targeted or complete genomes [123].

Besides characterizing genetic variants, it is worthwhile to study epigenetic changes, as epigenetic modifications can be influenced by external factors (e.g., lifestyle factors or intervention). If an individual is diagnosed with a particular genetic variant associated with a specific disease, epigenetic changes may be useful to control or switch off the expression of the specific gene through DNA methylation. By understanding both genetic variants and epigenetic changes in an individual or population, it is possible to offer a more targeted treatment which may potentially reduce the disease occurrence.

Bisulfite conversion with or without PCR amplification is generally used for methylation and mutation sequencing. In methylation sequencing, incomplete conversion of unmethylated cytosine to uracil followed by PCR amplification can lead to overestimation of methylation levels [124]. For mutation sequencing or other applications, biases may arise when short sequences or those with extreme GC content are preferentially or non-preferentially amplified [125]. Although reducing PCR cycles has been proposed as a strategy to minimise bias, research has shown limited improvement with an even lower correlation [125].

While methods such as bisulfite sequencing and methylated DNA immunoprecipitation (MeDIP) are commonly used to study epigenetic modifications, recent advances have enabled PCR-free sequencing approaches that bypass the need for bisulfite conversion. Platforms such as Illumina and Oxford Nanopore have been used for mtDNA sequencing, each offering distinct advantages and limitations (Table 3). Although PacBio technology has the potential to sequence full-length DNA without involving PCR amplification and bisulfite treatment, its application in detecting mtDNA methylation remains limited and underexplored in the current literature.

**TABLE 3:** Sequencing methods used for the detection of epigenetic modifications in mtDNA.

Sequencing methods	Epigenetic mark detected	PCR/Bisulfite conversion	Advantages	Limitations	References
Illumina	5-mC	Yes	- Used mtDNA-specific primer sets to exclude nuclear mtDNA segments	- Required fragmented DNA. - Introduced bias due to bisulfite conversion and PCR amplification	[126]
	5-mC	Yes			[127]
	5-mC	Yes			[128]
Nanopore sequencing	5-mC	No	- Enabled direct sequencing of linearised long read mtDNA or gDNA. - Excluded possible nuclear mtDNA segments from analysis - Overcame bias introduced by bisulfite conversion and PCR amplification - Detected both 5-mC and 6mA methylation marks	- Required high read depth to accurately detect methylation, increasing the cost	[96]
	5-mC	Both PCR-amplified and native mtDNA were used			[129]
	5-mC (applicable for 6mA)	Long-range PCR was used			[130]
	5-mC	No			[131]
Pyrosequencing	5-mC	Yes	- Offered lower cost, suitable for validation studies - Provided precise quantification (%) of methylation at specific CpG sites	- Introduced bias due to bisulfite conversion and PCR amplification - Limited to short, targeted reads	[127]
	5-mC	Yes			[132]
	5-mC	Yes			[133]
	5-mC	Yes			[134]
PacBio single molecule real-time sequencing	6mA	PCR was used	- Enabled direct sequencing of mtDNA or gDNA. - Overcame bias introduced by bisulfite conversion - Detected both 5-mC and 6mA methylation marks	- Potentially misidentified 5-mC as 6mA - Introduced bias due to PCR amplification	[135]

### Data Analysis and Validation

After a successful sequencing run, the large volume of sequencing data requires extensive bioinformatics expertise for analysis to identify significant mutations or epigenetic changes. The computational analysis typically involves three essential steps: (1) Data processing and quality control to ensure accuracy, (2) Data visualisation and statistical analysis to identify patterns and trends, and (3) Validation and interpretation to confirm findings and assess their significance [136]. While minimising error rates remains a key objective for reliable results, ongoing optimisation is still required for different sequencing methods. Despite the availability of various analytical tools, a clear guideline for analysis settings and thresholds has yet to be established [136]. Different studies have adopted different approaches, often employing multiple software tools at different stages [137]. This lack of standardisation poses significant challenges in sequencing analysis. Additionally, comparing results across studies using different analysis methods can further complicate data interpretation.

### Ethical Issues

Gene sequencing can reveal extensive genetic information, including adverse functional alleles of protein-coding genes and private individual variations that can be used to identify patients or their close relatives [138]. This raises ethical dilemmas regarding the disclosure of results to the individual or their relatives [139]. While increased awareness of potential hereditary conditions may promote healthier lifestyles, it can also lead to excessive anxiety or detrimental effect on individuals’ perspectives on their health and psychological wellbeing [140, 141].

In cases of maternally inherited mitochondrial diseases, caused by mtDNA mutations, several alternative strategies have been suggested to reduce or prevent the transmission of mtDNA from mother to child. These strategies include egg donation, prenatal testing, preimplantation genetic diagnosis and mitochondrial donation, all raising ethical concerns. Egg donation results in the child being genetically related to only one biological parent without the mitochondrial disease. Prenatal testing and preimplantation genetic diagnosis may risk the pregnancy and bring additional emotional burden on parents in deciding on whether to continue or prematurely terminate the pregnancy, if mitochondrial disease is detected [142]. Mitochondrial donation may be a more permissible approach as only the child’s mtDNA is replaced with mtDNA from a healthy donor while the nuclear DNA remain from both the biological parents [142]. However, concerns remain regarding the technical practicalities of complete faulty mtDNA replacement, as well as potential mismatches between the mtDNA haplotypes of the biological and donor mothers [142].

## Conclusions

The increasing prevalence of T2DM in recent decades, along with its future projections and prevalence have raised global concerns. In response, numerous initiatives have been introduced worldwide to promote healthier lifestyles and lower the risk of T2DM. However, while lifestyle factors can have a significant impact on T2DM development, it is also essential to acknowledge the significant influence of inherited genetic factors. Although these genetic predispositions are difficult to alter, epigenetic mechanisms may offer a potential means to regulate harmful gene expression patterns that may contribute to the increased risk of developing T2DM.

This review highlights the emerging roles of mtDNA mutations and epigenetics in the pathogenesis of T2DM. Evidence suggests that alterations in mitochondrial gene expression and function may significantly impair metabolism, leading to T2DM. Given the interplay between inherited genetic predisposition and epigenetic regulation, future research should prioritise large-scale clinical trials to investigate these relationships and susceptibility to T2DM in diverse populations. By integrating genetic background and epigenetic modifications, a more advanced personalised treatment for T2DM could be developed to prevent the development and progression of T2DM.
